# Supplementary material for: Splice-Junction-Based Mapping of Alternative Isoforms in the Human Proteome
Source: Cell Rep. Author manuscript; Available in PMC 2020 Jan 15. (PMC6961840; doi:10.1016/j.celrep.2019.11.026)
Supplement: 2 [file NIHMS1546469-supplement-2.zip › DF1/PXD006675/RightAtrium/RightAtrium_45_SARM1_RNSGSQLARNATLPAALYWWQTQWGHSR.pdf]

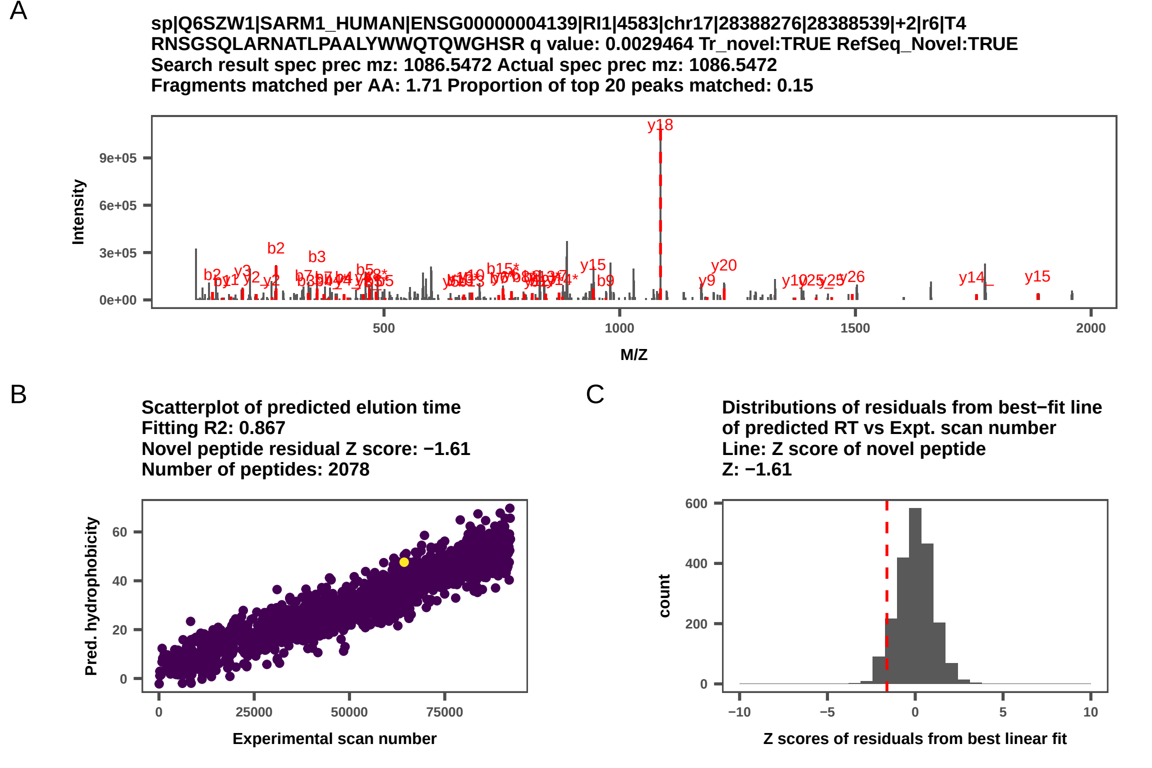
A
sp|Q6SZW1|SARM1_HUMAN|ENSG00000004139|RI1|4583|chr17|28388276|28388539|+2|r6|T4
RNSGSQLARNATLPAALYWWQTQWGHSR q value: 0.0029464 Tr_novel:TRUE RefSeq_Novel:TRUE
Search result spec prec mz: 1086.5472 Actual spec prec mz: 1086.5472
Fragments matched per AA: 1.71 Proportion of top 20 peaks matched: 0.15
Intensity
9e+05
6e+05
3e+05
0e+00
y18
b2
b3
y15
b9
y9
y20
y26
y14_
y15
500
1000
1500
2000
M/Z
B
Scatterplot of predicted elution time
Fitting R2: 0.867
Novel peptide residual Z score: −1.61
Number of peptides: 2078
Pred. hydrophobicity
60
40
20
0
0
25000
50000
75000
Experimental scan number
C
Distributions of residuals from best−fit line
of predicted RT vs Expt. scan number
Line: Z score of novel peptide
Z: −1.61
count
600
400
200
0
−10
−5
0
5
10
Z scores of residuals from best linear fit
